# Immunotherapy to Avoid Orbital Exenteration in Patients With Cutaneous Squamous Cell Carcinoma

**DOI:** 10.3389/fonc.2021.796197

**Published:** 2022-01-18

**Authors:** Luke S. McLean, Annette M. Lim, Angela Webb, Karda Cavanagh, Alesha Thai, Matthew Magarey, Carly Fox, Stephen Kleid, Danny Rischin

**Affiliations:** ^1^ Department of Medical Oncology, Peter MacCallum Cancer Centre, Melbourne, VIC, Australia; ^2^ Sir Peter MacCallum Department of Oncology, Faculty of Medicine, Dentistry and Health Sciences, The University of Melbourne, Melbourne, VIC, Australia; ^3^ Department of Plastic Surgery, Peter MacCallum Cancer Centre, Melbourne, VIC, Australia; ^4^ Department of Cancer Imaging, Peter MacCallum Cancer Centre, Melbourne, VIC, Australia; ^5^ Department of Head and Neck Surgery, Peter MacCallum Cancer Centre, Melbourne, VIC, Australia

**Keywords:** immunotherapy, cutaneous squamous cell carcinoma, orbital exenteration, anti-PD1, pseudoprogression

## Abstract

**Background:**

Cutaneous squamous cell carcinoma (CSCC) of the head and neck can require complex and disfiguring surgery in order to achieve cure, which can be morbid and negatively impact patient quality of life. The management of advanced CSCC has been revolutionized by immunotherapy with current clinical trials also exploring its role in the neoadjuvant and adjuvant settings. Patients may decline morbid curative surgery, such as orbital exenteration, and the outcomes of immunotherapy use in this unique group of patients require further investigation.

**Methods:**

We reviewed the records of 119 patients treated at a major Australian quaternary oncology centre with immunotherapy (either cemiplimab or pembrolizumab) for advanced CSCC.

**Results:**

We identified 7 patients recommended curative surgery involving orbital exenteration after multidisciplinary discussion, who declined surgery due to concerns about morbidity and/or disfigurement. All 7 patients demonstrated a response to treatment, and six avoided orbital exenteration. Two patients experienced pseudoprogression.

**Conclusions:**

The management of CSCC can be complex and requires the input of a multidisciplinary team. Immunotherapy to avoid or reduce the extent of morbid definitive surgery is an emerging treatment option.

## Introduction

Cutaneous squamous cell carcinomas (CSCC) are among the most commonly diagnosed malignancies in the United States of America (USA) and Australia (1). The majority of patients present with localized disease for which surgical resection is curative; however, approximately 5% of patients present with locoregionally advanced disease (2, 3). With a propensity for involvement of the head and neck in up to 80% of cases (4), there is a frequent need for complex and often disfiguring surgery with a significant impact on patient quality of life (5, 6). In an attempt to reduce the likelihood of recurrence for those with high-risk features, such as perineural involvement or positive margins, adjuvant radiotherapy is usually offered. However, despite aggressive upfront management, many patients will recur with advanced disease (7, 8).

The use of immunotherapy has revolutionized treatment for patients with unresectable or metastatic disease. Response rates of 50% with durable disease control have been reported resulting in regulatory approval in Europe and USA (9–12). The impressive responses are thought to be partly due to the high tumor mutational burden (TMB) of CSCC, which has correlated with improved response rates to immunotherapy in a number of different malignancies (13). A higher TMB is likely attributed to excessive ultraviolet exposure driving deoxyribonucleic acid (DNA) damage and a hypermutated phenotype for which there are more neoantigens to stimulate an effective anticancer immune response. Additionally, immunosuppression is associated with a 100-fold increased risk of CSCC development and particularly poor outcomes highlighting how closely tied this malignancy is with the immune system (14, 15).

The impressive response rates seen with immunotherapy in advanced CSCC, and evidence supporting a neoadjuvant application in other tumor types such as melanoma (16), have provided more confidence in usage in those patients who wish to avoid disfiguring or morbid curative surgery. The results of a recent phase II study exploring the role of neoadjuvant cemiplimab in patients with locoregionally advanced resectable head and neck CSCC yielded a high major pathological response rate of 70% (17). Other studies of neoadjuvant immunotherapy are ongoing. Literature and clinical trial data are lacking, however, for the role of immunotherapy in patients wishing to avoid potentially significant morbidity from surgery, a scenario that is increasingly encountered as immunotherapy access expands. We reviewed the medical records at our institute, a leading CSCC centre, for patients treated with immunotherapy in an attempt to avoid potentially curative, yet morbid, craniofacial surgery including orbital exenteration.

## Materials and Methods

This study was a single-center retrospective review of patients treated with immunotherapy for advanced CSCC after they declined definitive craniofacial surgery including orbital exenteration. Medical records between January 2016 and August 2021 were reviewed. For inclusion, patients must have been offered curative craniofacial surgery (including orbital exenteration) post multidisciplinary meeting discussion, who then subsequently declined surgery to pursue palliative immunotherapy. Patients with advanced CSCC who were receiving, or had received, immunotherapy included 7 patients who received pembrolizumab, 60 who had received compassionate access cemiplimab, and 52 who had received cemiplimab on clinical trial (NCT02760498). We identified 7 cases meeting our inclusion criteria. None of the 7 patients were involved in a clinical trial.

This study received approval from the Human Research Ethics Committee (HREC) of the Peter MacCallum Cancer Centre. Written consent was also received from the six patients who are currently alive, including for the use of all re-identifiable clinical photography.

## Results

### Case One

**Figure 1:**
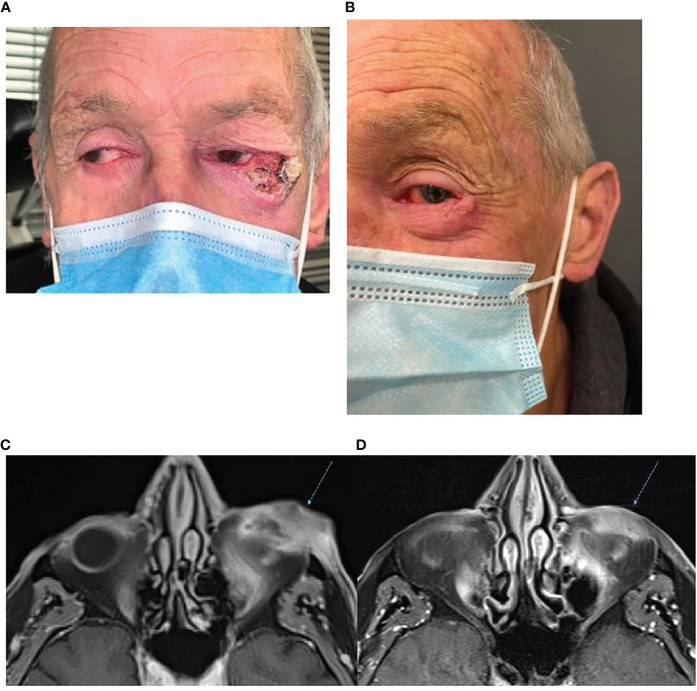
**(A)** Baseline photography of left lateral canthus CSCC prior to commencement of immunotherapy. **(B)** Photography of the lesion post two cycles of cemiplimab 350 mg/3-weekly demonstrating an excellent clinical response to therapy. **(C)** Baseline, left, MRI post-contrast T1 Fat Sat demonstrating 38 × 18-mm inferolateral canthus enhancing mass. **(D)** MRI post contrast T1 Fat Sat, post three cycles of cemiplimab 350 mg/3-weekly demonstrating reduced size and enhancement.

Immunotherapy has the ability to gain rapid oncological responses as was highlighted by Ferrarotto et al. in their neoadjuvant study where 70% of patients (14/20) achieved a complete or major pathological response post two cycles of cemiplimab 350 mg/3-weekly (17). Rapid clinical response is of particular importance for patients who have symptoms. Case one illustrates that immunotherapy use has the potential to gain both a rapid oncological and symptomatic response. A 66-year-old male presented with a large left lateral eyelid canthus lesion on the background of a previous left temple CSCC excision with adjuvant radiotherapy 4 years prior. Examination demonstrated an ulcerative lesion extending from the lateral canthus to involve the lateral half of the lid (**Figure 1A**) causing local irritation and diplopia. Magnetic resonance imaging (MRI) demonstrated that the lesion involved the left lateral rectus muscle and likely left inferior rectus muscles with broad abutment onto the left globe. Punch biopsy confirmed moderately differentiated CSCC. Fluorodeoxyglucose (*FDG*)-positron emission tomography (PET) imaging excluded distant disease. Vision was significantly impaired in his right eye secondary to glaucoma. He was offered curative surgery for the CSCC involving a left orbital exenteration which he declined due to his poor residual vision in his contralateral eye. He commenced cemiplimab 350 mg/3-weekly with an excellent clinical response reported post two cycles and complete resolution of his symptoms including diplopia (**Figure 1B**). Subsequent MRI post three cycles confirmed a response to treatment with the lesion having reduced in size and enhancement (**Figures 1C, D**). He has received a total of 7 cycles of cemiplimab currently with a sustained clinical and radiological response to treatment, complete resolution of pain and local irritation, and no reported immune-related adverse events (IRAEs).

### Case Two

**Figure 2:**
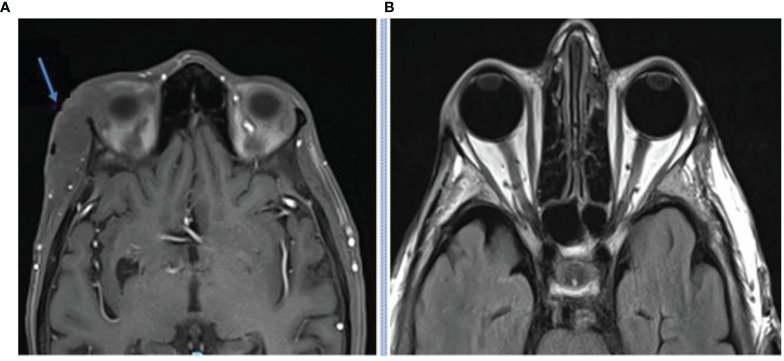
MRI imaging at baseline **(A)** and after 5 months of cemiplimab 350 mg/3-weekly **(B)** demonstrating resolution of the soft tissue orbital mass.

This case highlights the ability to use immunotherapy in an immunosuppressed patient on dialysis. Immunocompromised patients face a high risk of CSCC development including more aggressive disease and poorer treatment outcomes (15). Case two was a 59-year-old male with multiple comorbidities including end-stage renal failure secondary to a congenital unilateral kidney with ureteric malformations and a previous renal transplant 30 years prior complicated by graft rejection requiring nephrectomy and recommencement of hemodialysis. Even while off immunosuppressants, he had an extensive history of multiple CSCCs. He developed a 50-mm recurrent poorly differentiated CSCC of the right lateral orbit that recurred in an area of prior excision and radiotherapy completed 2 years earlier. He also had osteoradionecrosis of the skull from previous CSCC management for which he had declined neurosurgical intervention. FDG-PET identified the known CSCC of the right temple and lateral orbit as well as involvement of two level II right-sided lymph nodes. No distant metastases were identified. The multidisciplinary recommendation was for orbital exenteration and neck dissection which the patient deemed too morbid and high-risk in the setting of his comorbidities and opted for immunotherapy. He commenced cemiplimab 350 mg/3-weekly with treatment occurring separate to his dialysis days. MRI post 5 months of treatment demonstrated resolution of the soft tissue mass (**Figure 2**). He received a total of 12 months of immunotherapy without any reported IRAEs but died due to voluntary cessation of his dialysis.

### Case Three

**Figure 3:**
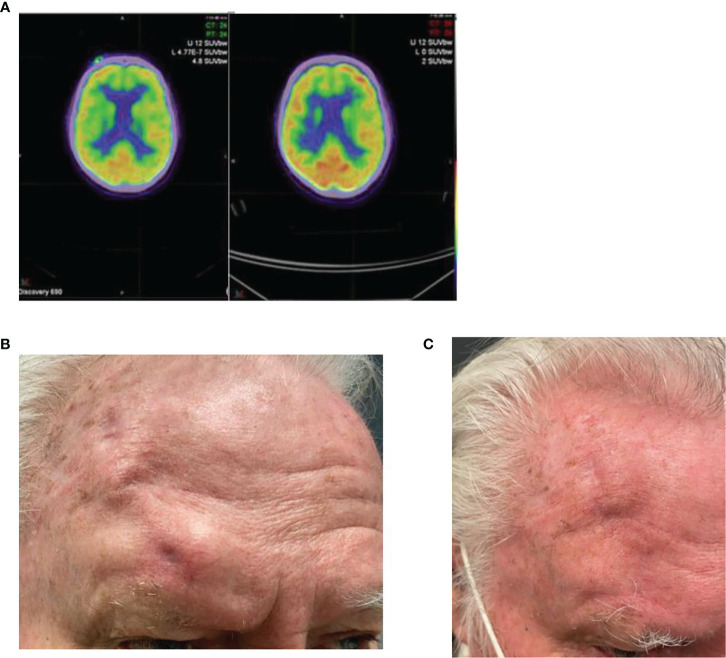
**(A)** Baseline FDG-PET imaging demonstrating an avid right supraorbital cutaneous malignancy with a complete metabolic response seen on follow up FDG-PET post four cycles of cemiplimab 350 mg/3-weekly. **(B)** Baseline photo of the right supraorbital clinical mass. **(C)** Post 7 cycles of cemiplimab demonstrating complete resolution.

This case highlights another example of orbital exenteration being avoided through a rapid response to immunotherapy. Case 3 is an 80-year-old male who was referred with a cutaneous mass at the right supraorbital region on a background of no significant past medical history. Biopsy confirmed poorly differentiated CSCC. MRI demonstrated two supraorbital lesions measuring 15 and 10 mm as well as enhancement of the supraorbital nerve consistent with perineural invasion. The proposed surgery consisted of a wide local excision, including excision of the involved nerve, plus orbital exenteration with expectant need for adjuvant radiotherapy. The patient declined curative surgery due to morbidity and elected to pursue cemiplimab 350 mg/3-weekly. FDG-PET imaging identified a moderately avid cutaneous malignancy in the right supraorbital region without any avid nodal involvement or distant disease. After four cycles of immunotherapy, repeat FDG-PET imaging demonstrated a complete metabolic response (**Figure 3**). He has tolerated therapy well with his only adverse event being subclinical hyperthyroidism which is being monitored without intervention to date.

### Case Four

**Figure 4:**
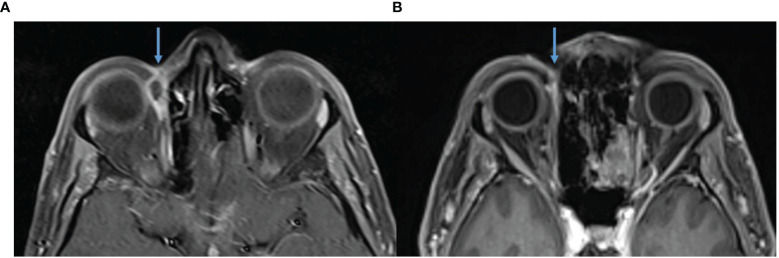
**(A)** Baseline, left, MRI post contrast T1 Fat Sat demonstrating 18 × 9 mm right medial extraconal orbital peripherally enhancing mass. **(B)** MRI post contrast T1 Fat Sat, post five cycles of pembrolizumab 200 mg/3-weekly, no mass like enhancement.

This case demonstrates the potential for a long-term durable response to immunotherapy and highlights a potential curative role for immunotherapy in these patients. A 45-year-old male presented with a right superomedial extraconal orbit mass in the context of having had a cutaneous microcytic adnexal carcinoma excised in the same region 5 years prior, followed by adjuvant radiotherapy. MRI demonstrated an 8-mm lesion within the medial right orbit and involvement of the superior right orbit (**Figure 4**) and FDG-PET scan demonstrated focal low-grade uptake in the lesion with no other avid sites of disease. Biopsy revealed squamous cell carcinoma. He had superior orbital nerve paraesthesia, without obvious enhancement on MRI, but was otherwise asymptomatic. He was offered orbital exenteration and frontoethmoidectomy with partial maxillectomy and a free flap reconstruction. He however declined surgery and received pembrolizumab 200 mg/3-weekly for 12 months. FDG-PET imaging post 12 months of immunotherapy demonstrated a complete metabolic response, and ongoing surveillance MRI to date has been clear 4 years and 5 months since his recurrence (**Figure 4**). He tolerated therapy well with no IRAEs.

### Case Five

**Figure 5:**
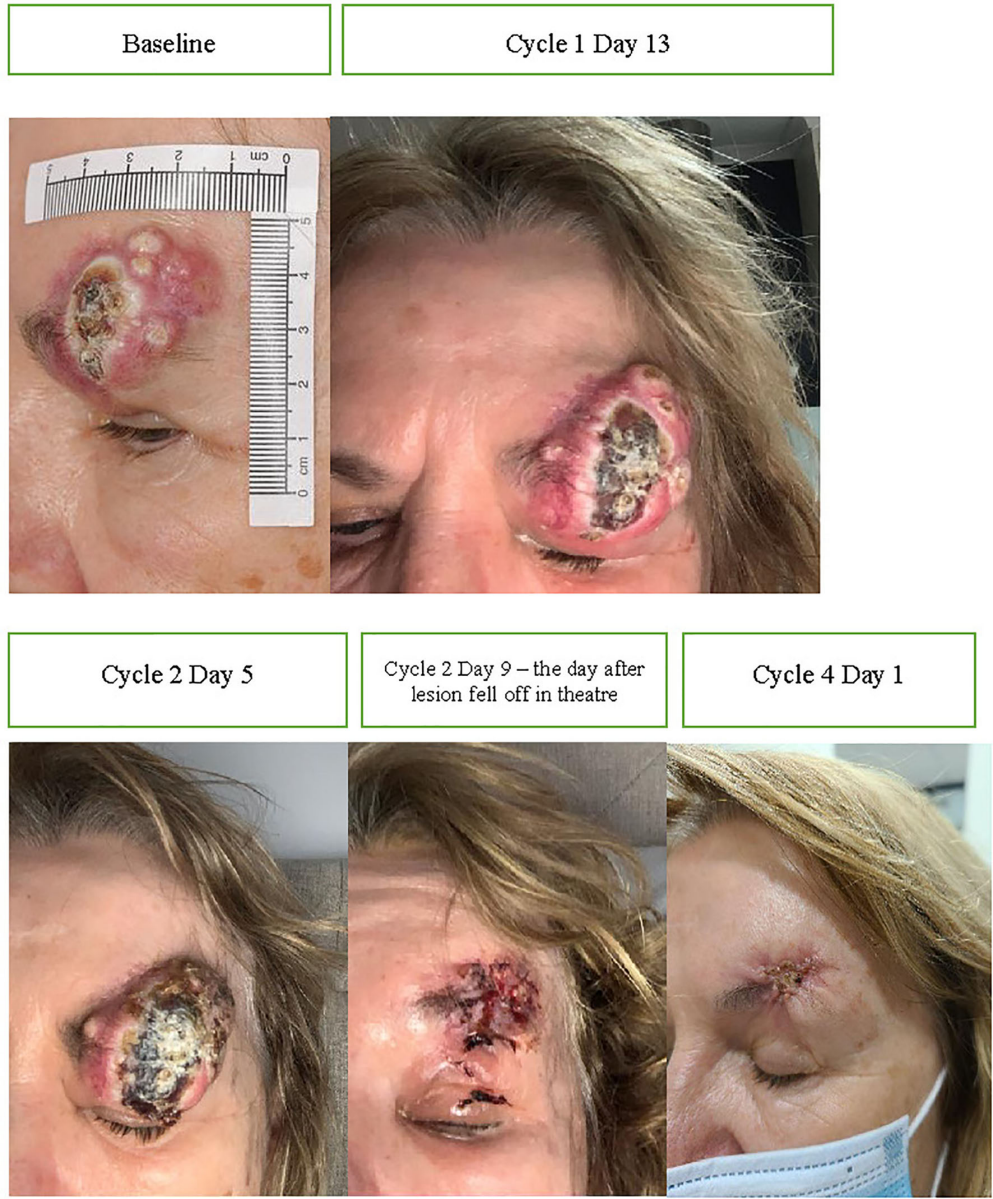
Baseline images of CSCC and throughout initial treatment demonstrating enlargement post commencement of cemiplimab 350 mg/3-weekly (consistent with pseudoprogression) and improvement after the lesion fell at off the time of surgery.

**Figure 6:**
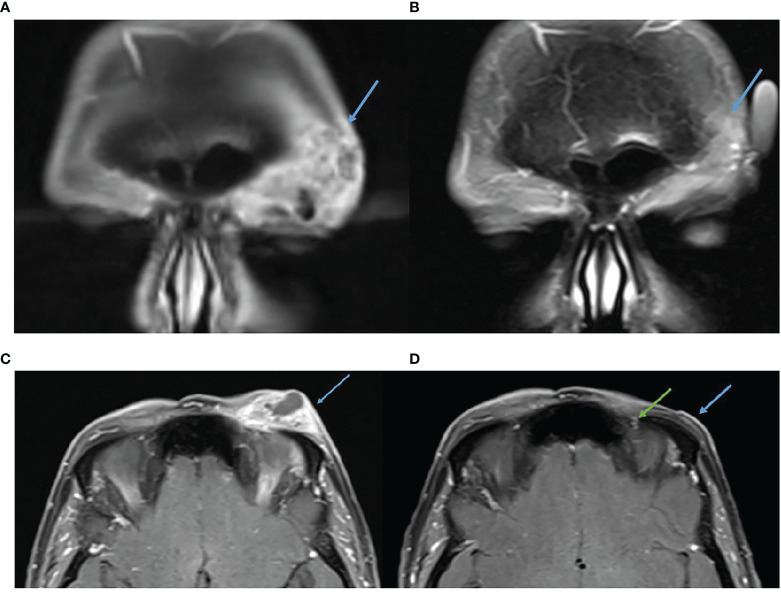
**(A)** Baseline, left, MRI post contrast T1 Fat Sat demonstrating 39 × 16 mm left forehead enhancing mass. **(B)** MRI post contrast T1 Fat Sat, post 16 cycles of cemiplimab 350 mg/3-weekly, demonstrating response. **(C)** Baseline, left, MRI post contrast T1 Fat Sat demonstrating 39 × 16 mm left forehead enhancing mass. **(D)** MRI post contrast T1 Fat Sat, post 16 cycles of cemiplimab 350 mg/3-weekly, no mass like enhancement and only small volume residual enhancement of the left supraorbital nerve, green arrow.

This case highlights an example of pseudoprogression followed by a rapid response shortly after the commencement of immunotherapy. A 65-year-old female with no significant past medical history presented with a large left supraorbital CSCC with numbness in the distribution of the ophthalmic division of the trigeminal nerve. This occurred in the context of having a well-differentiated CSCC excised 1 month prior with positive margins. Biopsy confirmed CSCC. MRI demonstrated a 39 × 16 × 19-mm lesion with thickening and enhancement of the left supraorbital nerve. FDG-PET excluded distant metastatic disease but identified bilateral cervical and mediastinal adenopathy for which sarcoidosis was confirmed after biopsy. The multidisciplinary team recommended orbital exenteration and adjuvant radiotherapy. This was declined by the patient, and immunotherapy was pursued with cemiplimab 350 mg/3-weekly. Post commencement of immunotherapy, the lesion became larger causing complete left sided ptosis (**Figure 5**) and after a further dose, she eventually consented to craniofacial surgery due to concerns of progression. During skin preparation in theatre, the lesion fell off and after discussion with the next of kin surgery was delayed to permit pathological assessment. Histopathology demonstrated necrotic tissue with no viable tumor consistent with a complete pathological response. There were also features of inflamed tissue, including granulation tissue, thought to support a clinical picture of pseudoprogression. Following multidisciplinary consensus, ongoing immunotherapy was recommended. Post four cycles of immunotherapy, there was significant improvement in the findings on MRI including near resolution of thickening and enhancement of the left superior orbital nerve and in the enhancement of the soft tissues over the left forehead (**Figure 6**). To date, she continues on cemiplimab post 18 cycles with an ongoing response. Treatment has also been well tolerated with no IRAEs.

### Case Six

**Figure 7:**
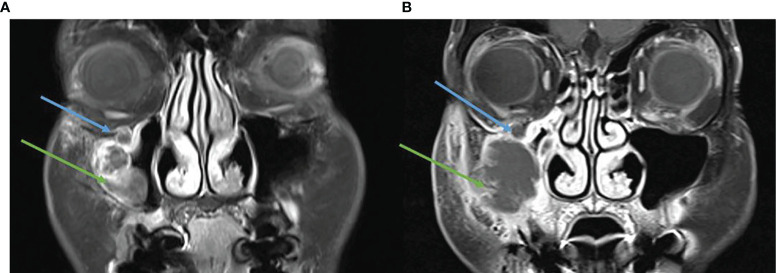
**(A)** Baseline, left, MRI post contrast T1 Fat Sat demonstrating a destructive lesion involving the anterior wall of the right maxillary sinus (green arrow) and perineural spread in the right infraorbital nerve (blue arrow). **(B)** MRI post contrast T1 Fat Sat, post three cycles of cemiplimab 250 mg/3-weekly, enlarging destructive mass and persisting perineural spread.

Case six also demonstrates pseudoprogression and highlights the complexity of immunotherapy response assessment in CSCC patients. In particular, it demonstrates an example of poor correlation between imaging and pathological response rates. A 68-year-old female presented with painful recurrent CSCC of the right maxilla extending along the right infraorbital nerve. She had a prior right cheek SCC *in situ* managed with topical fluorouracil 2 years prior. The multidisciplinary recommendation was for surgery including maxillectomy, partial rhinectomy, excision of the involved nerve, and free flap repair. The patient had significant concerns about the morbidity and disfigurement of surgery and declined curative surgery. She commenced cemiplimab 350 mg/3-weekly without adverse events and with initial improvement in symptoms with improved eyelid mobility. However, after four cycles the lesion was noted to be rapidly enlarging. Given initial symptomatic improvement, and concerns this enlargement could represent pseudoprogression, a fine-needle aspirate (FNA) was performed of the right cheek lesion. This demonstrated abundant keratinized squamous cells with a small amount of necrotic inflammatory debris. The aspirated material did not facilitate a diagnosis of malignancy nor was it possible to determine whether the aspirate represented necrotic material. MRI demonstrated the primary CSCC lesion had increased from 41 × 26 mm to 52 × 51 mm, with multifocal areas of progression and new extension to the anterior maxillary wall (**Figure 7**). FDG-PET showed uptake on the anterior margin of the tumor, whereas the bulk of the mass now appeared cystic. Despite use of multiple imaging modalities and aspirate to exclude pseudoprogression, it remained difficult to determine. The patient reported some worsening of paresthesia along the upper lip and increasing pressure sensation of the right cheek. After multidisciplinary discussion and review of the patient, she consented to a right maxillectomy, partial rhinectomy, orbital exenteration, neck dissection, and free flap. Histopathology demonstrated extensive regression characterized by extensive residual keratin with no viable tumor seen in maxilla, maxillary sinus, or orbital contents. Residual viable 52 × 33 × 24-mm moderately differentiated SCC in soft tissue deep to subcutis was completely excised. Neck dissection demonstrated absence of tumor in 22 lymph nodes. Following multidisciplinary discussion, cemiplimab was discontinued after surgery. Due to the extensive tumor at diagnosis, and presence of some residual tumor after abandoned immunotherapy, the patient subsequently received adjuvant radiotherapy (60 gray in 30 fractions).

### Case Seven

**Figure 8:**
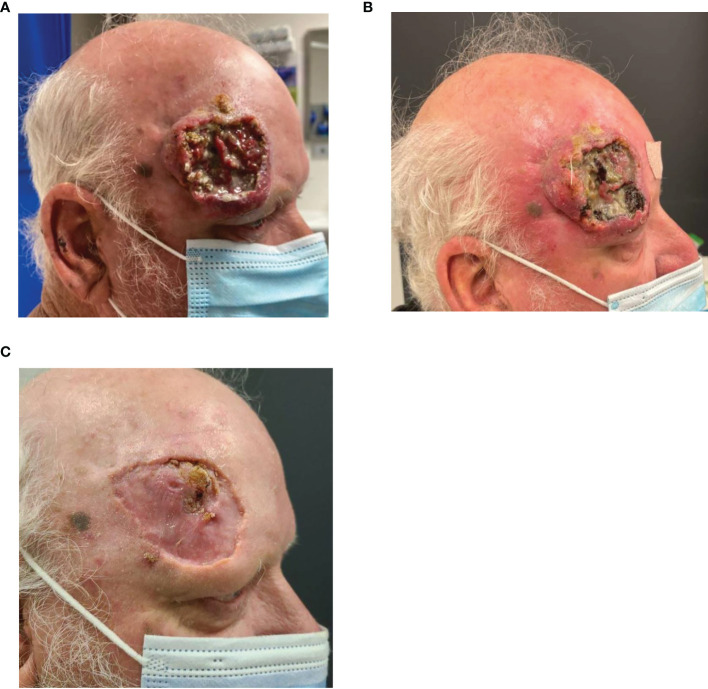
**(A)** Baseline images of right forehead CSCC. **(B)** Improvement noted post two cycles of cemiplimab 350 mg/3-weekly and **(C)** further improvement noted again post cycle 10.

Case seven is an example of a patient with a fungating and disfiguring primary tumor who has demonstrated an excellent clinical response to immunotherapy. The patient is a 63-year-old man with a locally extensive CSCC of the right forehead on a background of no significant past medical history. MRI demonstrated a large ulcerated 60-mm lesion contacting the calvarium, extending to the lateral aspect of the upper eyelid with associated edema extending into the lateral preseptal tissue and lacrimal gland (**Figure 8A**). FDG-PET demonstrated the right scalp malignancy with likely nodal metastasis in a right pre-auricular node and avid level II node. FNA of the intraparotid and cervical nodes, however, confirmed reactive changes. Multidisciplinary review confirmed lacrimal gland and post-septal involvement and a recommendation for craniofacial surgery including orbital exenteration was made. The patient declined this procedure and commenced cemiplimab 350 mg/3-weekly. He demonstrated a rapid clinical response noted post two cycles of treatment (**Figure 8B**). He currently maintains a good response to cemiplimab post 10 cycles and has experienced no IRAEs (**Figure 8C**).

## Discussion

Factors such as treatment morbidity, goals of therapy, comorbidities, and patient preference are all essential elements to consider when offering oncological therapy. Locally advanced CSCC of the head and neck can be disfiguring and fungate resulting in a significant functional and cosmetic impact to the patient that is often then further exacerbated by the extensive surgical procedures used to control it. This distinct “real-world” cohort of vulnerable patients had disease in the functionally and cosmetically sensitive periorbital region who declined orbital exenteration and who mostly had excellent outcomes with immunotherapy. While surgical intervention with or without adjuvant radiotherapy remains the mainstay of cure for these patients with locally advanced disease, there are a number of patients who deem the morbidity of such interventions too significant and opt for immunotherapy. Disease control with immunotherapy can be durable, approaching 5 years as demonstrated by case four. In metastatic melanoma, the 5-year progression-free survival for combination ipilimumab/nivolumab and nivolumab alone is 52% and 44% respectively, with median duration of response not yet reached (18). While it is too early for the 5-year survival outcomes from the CSCC immunotherapy studies, durable responses as seen in case four may represent a curative potential for immunotherapy in CSCC. Despite the potential for a deep response to immunotherapy, we do not currently know how to identify which patients with advanced CSCC will achieve this level of benefit. There is also some uncertainty about the durability of responses which will be answered with longer trial follow-up.

Rapid oncological responses can be seen with immunotherapy, which is of great appeal when rapid disease control is required for symptom control or to preserve critical organ function. The KEYNOTE-629 study using pembrolizumab 200 mg/3-weekly demonstrated a median time to response of 1.5 months (95% confidence interval (CI) 1.2 to 5.7) with an early improvement seen for a number of large fungating tumors (11). Similar results were also seen in the pivotal cemiplimab study with a median time to response of 2.3 (95% CI 1.7–7.3) and 1.9 (1.7–6.0) months in the phase 1 expansion and phase 2 cohorts respectively (9). It should be noted, however, that the time to clinical response reflects the timing of that assessment or treatment visit schedule rather than the first time point at which responses can be observed. The health-related quality of life analysis of the phase 2 cemiplimab study has since demonstrated a median time to first clinically meaningful improvement in pain of 2.1 months (2.0–3.7 months) that was also durable (19). Due in part to the location of these tumors in the head and neck and a tendency for perineural involvement, symptom control remains an important component of patient quality of life. The potential for immunotherapy to achieve this was highlighted in case one with a complete resolution of symptoms post two cycles of cemiplimab. Additionally, all of the described cases emphasize that the avoidance of orbital exenteration was of critical importance to patient quality of life, a procedure that was ultimately avoided in six of the described cases.

Two of the described cases exhibited pseudoprogression. Reports on the incidence of pseudoprogression vary, but it is generally thought to occur in less than 10% of all cancers treated with immunotherapy (20–24). Both of these cases demonstrated features of radiological and clinical progression resulting in patients accepting surgical intervention, after which histopathological review demonstrated a complete pathological response in case five and only a small area of residual moderately differentiated CSCC in case six. The increase in tumor size seen both on imaging and clinically likely reflected an increased inflammatory cell infiltrate followed by treatment response (25, 26). Immune-specific response criteria have subsequently been developed to help address pseudoprogression but are yet to be validated prospectively in advanced CSCC (27–30). For example, the Response Evaluation Criteria in Solid Tumors (RECIST) has been modified for immune-based therapeutics (termed iRECIST), taking into account the potential for this phenomenon. In cases of suspected pseudoprogression, treatment beyond “progression” only continues if the patient’s performance status and symptoms remain stable and repeat imaging is required after a further 4 weeks to confirm whether true progression has occurred or not. However, as described in the example of case five, limitations exist. In this case, pseudoprogression resulted in an enlarging supraorbital mass causing new complete left sided ptosis. Case six represents a similar scenario where FNA was arranged to aid decision making but was unable to differentiate between true progression versus pseudoprogression. While the concept of collecting tissue to review whether there is a predominance of underlying malignant squamous cells or features of treatment response (such as the presence of immune cells, granulation, tumor regression) is of sound rationale, adequate sampling and access to all sites of disease in an esthetically complex region such as the periorbital region are often not possible. Similarly, there is no standardized approach as to when the most optimal timing for tissue collection may be, what technique should be used, or how the histopathological findings should impact the overall treatment approach. Given the complete pathological response that occurred in case five, one must also consider whether further immunotherapy in the example of case six would also have resulted in a complete pathological response. Had this occurred, the subsequent clinical and radiological improvements may have resulted in her avoiding extensive surgery. However, the optimal number of immunotherapy cycles in this population is yet to be defined. The traditional assessments of tumor response and when to abandon therapy for patients with functional or impending functional consequences due to enlarging disease while on immunotherapy remain inadequate and warrant further research.

The only published neoadjuvant cemiplimab study utilizing cemiplimab 350 mg/3-weekly with a response assessment post two cycles followed by surgery demonstrated a remarkable 70% major pathological response rate with 55% of patients achieving a complete pathological response (17). All patients in this study received curative surgery as part of the study design; however, these impressive results raise the pertinent question of whether less extensive surgery is feasible following immunotherapy, a potential area of investigation for future studies. With the caveat that this was a small cohort of 20 patients, they also identified that there was poor correlation between imaging and pathological response rates supporting a growing body of evidence that RECIST criteria may be underestimating the number of true treatment responses (17, 31). A salient finding was that 60% of patients were able to avoid adjuvant radiotherapy due to their treatment response, an intervention that was recommended for all at time of enrolment (17). With impressive pathological response rates and the potential to avoid radiotherapy and associated morbidity, a neoadjuvant treatment pathway is a focus of ongoing clinical research.

As demonstrated by these cases, responses to immunotherapy can be rapid with cases one, five, six, and seven demonstrating a clinical and radiological response within a few cycles of immunotherapy and case four demonstrating the potential for such responses to translate into long-term durable disease control. In the pooled analysis of the phase II cemiplimab study in the advanced setting, there was note of an increase in complete response rates over time from 9% to 16%, and median overall survival and duration of response were not reached, supporting this observation (10). Ongoing research is investigating the role of immunotherapy in both the neoadjuvant and adjuvant settings; however, the described cases in this article represent a unique group of patients with advanced resectable disease who declined surgical intervention, a group for which there are currently no prospective data on immunotherapy outcomes and for whom the design of a randomized study would not be feasible or ethical. Additionally, the responses described in this series highlight the need for further prospective research into biomarkers that will allow clinicians to identify patients who are most likely to derive benefit from immunotherapy in this setting. As immunocompromised patients face higher rates of toxicity (including the risk of allograft rejection in solid organ transplant recipients), predictive biomarkers to help balance discussions around treatment benefit versus risk are even more critical in this group. In other solid cancers, immunotherapy response has been associated with immunological factors such as the presence of cytotoxic CD8^+^ T cell populations and expression of checkpoint ligands (i.e., programmed death-ligand 1, PD-L1) as well as tumor intrinsic factors such as TMB; however, this has not been established in CSCC cohorts. The functional and cosmetic impacts of this malignancy and its curative treatment strategies are important to recognize, and the potential benefits and limitations of immunotherapy in this setting should be understood by medical oncologists, radiation oncologists, imaging specialists, and surgeons alike who all play a vital role in the multidisciplinary care of these complex patients.

## Data Availability Statement

The original contributions presented in the study are included in the article/supplementary material. Further inquiries can be directed to the corresponding author.

## Ethics Statement

The studies involving human participants were reviewed and approved by the Peter MacCallum Cancer Centre Ethics Committee. The patients/participants provided their written informed consent to participate in this study. Written informed consent was obtained from the individual(s) for the publication of any potentially identifiable images or data included in this article.

## Author Contributions

Concept and design—LM, AML, AW, DR. Collection and assembly of data—LM, KC. Data analysis and interpretation—all authors. Manuscript writing—all authors. Final approval of manuscript—all authors. All authors contributed to the article and approved the submitted version.

## Funding

DR is supported by a NHMRC Investigator Grant APP1175929.

## Conflict of Interest

AML uncompensated advisory board from Merck Sharp & Dohme and Bristol-Myers Squibb with travel and accommodation expenses; uncompensated consultancy for Eisai. DR receives institutional research funding from: MSD, GSK, BMS, Roche, Replimune, Kura Oncology, Decibel, Regeneron, Sanofi, Merck KGaA. Trial Steering Committees and/or Advistory Boards (all uncompensated): MSD, GSK, Regeneron, Sanofi.

The remaining authors declare that the research was conducted in the absence of any commercial or financial relationships that could be construed as a potential conflict of interest.

## Publisher’s Note

All claims expressed in this article are solely those of the authors and do not necessarily represent those of their affiliated organizations, or those of the publisher, the editors and the reviewers. Any product that may be evaluated in this article, or claim that may be made by its manufacturer, is not guaranteed or endorsed by the publisher.
